# Development of microsatellite markers for sister species *Linum suffruticosum* and *Linum tenuifolium* in their overlapping ranges

**DOI:** 10.1007/s11033-023-08471-9

**Published:** 2023-07-17

**Authors:** Erika Olmedo-Vicente, Aurélie Désamoré, Violeta I. Simón-Porcar, Tanja Slotte, Juan Arroyo

**Affiliations:** 1 Department of Plant Biology and Ecology, University of Seville, Seville, Spain; 2 Department of Ecology, Environment and Plant Sciences, Science for Life Laboratory, Stockholm University, Stockholm, Sweden

**Keywords:** Heterostyly, Floral polymorphism, Genetic variation, *Linum*, Microsatellites, Hybrid zones

## Abstract

**Background:**

Microsatellite markers were developed for distylous *Linum suffruticosum* and tested in the monomorphic sister species *Linum tenuifolium*. These species are perennial herbs endemic to the western and northwestern Mediterranean, respectively, with a partially overlapping distribution area.

**Methods and results:**

We developed 12 microsatellite markers for *L. suffruticosum* using next generation sequencing, and assessed their polymorphism and genetic diversity in 152 individuals from seven natural populations. The markers displayed high polymorphism, with two to 16 alleles per locus and population, and average observed and expected heterozygosities of 0.833 and 0.692, respectively. All loci amplified successfully in the sister species *L. tenuifolium*, and 150 individuals from seven populations were also screened. The polymorphism exhibited was high, with two to ten alleles per locus and population, and average observed and expected heterozygosities of 0.77 and 0.62, respectively.

**Conclusions:**

The microsatellite markers identified in *L. suffruticosum* and tested in *L. tenuifolium* are a powerful tool to facilitate future investigations of the population genetics, mating patterns and hybridization between both *Linum* species in their contact zone.

**Supplementary Information:**

The online version contains supplementary material available at 10.1007/s11033-023-08471-9.

## Introduction

*Linum* L. (Linaceae) is a cosmopolitan and diverse genus with a great economic and ecological importance. In addition, it stands as a model system for studying the evolution of heterostyly from the early observations of Darwin [1] to the last advances on genomics of the S-locus [2]. Heterostyly consists in the co-occurrence of two to three floral morphs within a population, with floral morphs (1) being hermaphroditic, and (2) presenting stigmas and anthers at different reciprocal positions within the flower [3]. *Linum* exhibits high variation in morphology, mating system and presence of heterostyly and related floral polymorphisms, which have evolved multiple independent times [4-6]⁠.

**Fig. 1:**
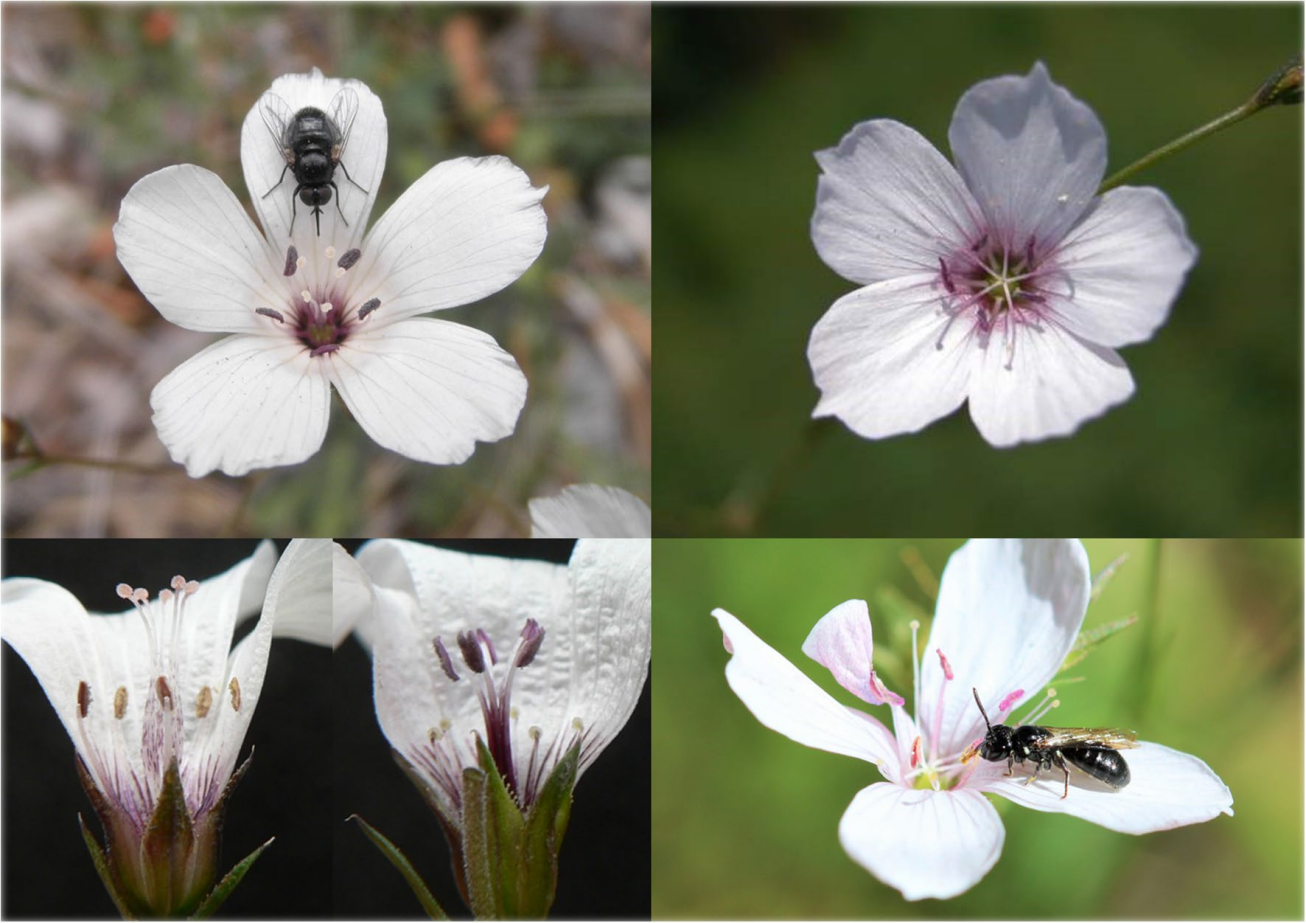
Flowers of distylous *Linum suffruticosum* (left) and style-monomorphic *Linum tenuifolium* (right), with details of their sex organs and two common pollinators

The sister species *Linum suffruticosum* and *L. tenuifolium* (Fig. 1) appear as an ideal study system to assess the microevolutionary mechanisms that support the maintenance and loss of heterostyly in *Linum* [7, 8]. Distributed in the western Mediterranean Basin, *Linum suffruticosum* is a heteromorphic and self-incompatible species showing a unique case of three-dimensional heterostyly [8]. The self-compatible and monomorphic *L. tenuifolium* is the sister species of *L. suffruticosum* and is distributed in southern Europe [6, 9, 10]. Both species have a contact zone area in the NW of the Mediterranean Basin, from NE Spain to NW Italy, where populations co-occur in nearby sites or even intermingled and are able to hybridize [10]. ⁠This contact zone makes the *L. suffruticosum-L. tenuifolium* complex an excellent system to address questions about the evolution of mating systems, and to understand the processes underlying reproductive isolation and species divergence [11].

In the last twenty years, Simple Sequence Repeat markers (SSR) have been the most common tool for a variety of applications in molecular biology, from genome mapping to population and ecological genetics, due to their codominant mode of heredity and their highly polymorphic nature [12, 13]. SSR markers are commonly developed to investigate genetic variation within particular species [e.g. 14, 15]. However, SSR markers can be also transferable between closely related species when genomic resources are not available for *de novo* development [e.g. 16, 17].

To date, the development of molecular tools for population studies in *Linum* has been mostly restricted to the cultivated flax *L. usitatissimum* [18-20], meaning a lack of suitable molecular resources for studying the evolutionary ecology of several wild *Linum* species. Here, we characterize 12 new polymorphic microsatellite loci for *L. suffruticosum* and their transferability to *L. tenuifolium* in seven wild populations of each species. These markers will be useful for future research on the genetics, mating patterns as well as potential natural hybridization within and between sister species in their contact zone.

## Materials and methods

### Identification of candidate SSR loci and primer design

Genomic DNA was extracted from two individuals of *L. suffruticosum* sampled in a natural population (Prat d’Aguiló, Lleida, Spain; 42.34301, 1.71806) with Invisorb® Spin Plant Mini Kit. DNA was conveyed to Ecogenics GmbH (Schlieren-Zürich, Switzerland, https://www.ecogenics.ch) for the development of a library of suitable SSR candidates and primer design. The Illumina TruSeq Nano library was analyzed on an Illumina MiSeq sequencing platform with a nano v2 500 cycles sequencing chip. The chastity-filtered paired-end reads were subject to de-multiplexing and trimming of Illumina adapter sequences. Subsequently, the quality of the reads was checked with FastQC v0.117 software [21]. Afterwards, the paired-end reads were merged with the software USEARCH v10.0.240 [22]. The 99,943 merged reads were screened with the software Tandem Repeats Finder, v4.09 [23]. After this process, 5704 merged reads contained a microsatellite insert with a tetra- or a trinucleotide of at least six repeat units or a dinucleotide of at least ten repeat units. Primer design was performed with default parameters in Primer3 [24], resulting in 4243 microsatellite candidates.

### Primer testing and polymorphism assessment

A total of 302 individuals, from seven populations of each *L. suffruticosum* and *L. tenuifolium* distributed in their contact zone, were used for primer testing and polymorphism assessment (Online Appendix 1). Leaf tissue was collected from individuals separated at least 1 m from each other and preserved in silica gel. Vegetative reproduction is negligible or very limited in *L. tenuifolium* and *L. suffruticosum*, respectively. Some of the population sites were pure and other were mixed, containing both species that are clearly distinguishable (Fig. 1 and Online Appendix 1). *Linum tenuifolium* has been described as a diploid species throughout its range and, although *L. suffruticosum* is a polyploid complex, all populations screened were diploid [25].

We randomly selected 60 microsatellites to test their amplification in 2–4 individuals from each species and population (96 individuals in total). Genomic DNA was extracted with ISOLATE II Plant DNA Kit (Bioline). PCR amplifications were conducted using 20 µL of master mix that included: 1x MyTaq Red Reaction Buffer (Bioline), 0.4 μm of each forward and reverse primers, 0.01% bovine serum albumin (BSA, Promega), 0.5 u MyTaqTM Red DNA Polymerase (Bioline), 50–70 ng gDNA and deionized water up to 20 µL. A touchdown procedure was performed for all loci with initial denaturation for 2 min at 94 °C; followed by 10 cycles of 92 °C for 30 s, 30 s at 63 °C with an increment of − 1 °C per cycle, and 30 s at 72 °C; followed by 20 cycles of 94 °C for 30 s, 30 s at 56 °C, and 30 s at 72 °C; and an extra extension of 5 min at 72 °C. The amplification of PCR products was assessed in 2% agarose gels. Twelve markers that amplified well in both species (Table 1) were selected for polymorphism assessment.

**Table 1:** Characterization of 12 microsatellite loci identified in *Linum suffruticosum*

Locus	GeneBank accesion	Repeat motif	Repeat length	Primer sequence (5′ to 3′)	Amplicon size (bp)
**Ls_1145191**	OQ472634	TGA	10	F-GCTGCAAGTTCGACCTCC	116
				R-GCCGGTGATGATTTTCAGGG	
**Ls_1169143**	OQ472635	TG	18	F-CTCTGCACTTCTATTCCTGTAGC	158
				R-GCCTTGATCGGTCGCATAAC	
**Ls_1178187**	OQ472636	TTC	14	F-AATTCGTCAAGGAGGCAACG	189
				R-TGCCATTCAAAGGTAGTGAAAC	
**Ls_144692**	OQ472637	TTC	23	F-TCATCACCGTAACAAAGCCC	243
				R-GCCATTCAAAGGTGGTGAAAC	
**Ls_246481**	OQ472638	CAA	11	F-ATTGTTACTCGGCCACCCAC	103
				R-AAACGGGCATTGAACTTCGG	
**Ls_337128**	OQ472639	AG	25	F-CTCCTTTGATCTAGGCACGC	250
				R-GGCCAACTTCTAGCGACCG	
Ls_37372	OQ472640	AC	16	F-TGTATCAGTCGGGGGTTGAG	195
				R-CTCTGCACTTCTATTCCTGTAGC	
Ls_395648	OQ472641	AC	13	F-TCGTAGATTGGGGCGAGAAG	243
				R-TCTGCACTTCCATTCATGTAGC	
Ls_421659	OQ472642	GGA	8	F-TACGCAGAATGGTGGTTTGG	189
				R-AGTTTCATCGTTGTGGACGC	
Ls_807222	OQ472643	TTC	9	F-AAGATGTGCCCTCTCCATCC	173
				R-GAACCCTGCTTCTGGTTCAAG	
Ls_889692	OQ472644	GAA	14	F-TGCCATTCAAAGGTAGTGAAAC	192
				R-AATTCGTCAAGGAGGCAACG	
Ls_9438	OQ472645	GAA	24	F-TCAAATTGCCCAACAATTCTAGC	247
				R-AATTCGTCAAGGAGGCAACG	

Forward primers were labelled with either 6-FAM, VIC, NED or PET fluorescent labels for fragment analyses on 18–24 individuals from each species and population (302 individuals in total; Online Appendix 1). DNA extractions and PCR reactions were performed with the same protocol as for primer testing. PCR products were analysed on an automatic ABI 3730 capillary DNA sequencer (Sequencing Service, University of Dundee, UK), using a GeneScan 500 LIZ internal size standard. Allele binning and calling were performed in Geneious (Biomatters).

For each locus and population, the number of alleles per locus (*A*), observed heterozygosity (*H*_O_) and expected heterozygosity (*H*_E_) were calculated with the *popgenreport* function of R package PopGenReport [26]. The deviation from Hardy-Weinberg equilibrium for each locus was tested using the function *mk.hw*. The presence of null alleles was tested with the function *null*, following the methods of Brookfield [27] ⁠and Chakraborty et al. [28].

## Results and discussion

In this study, we characterized 12 SSR markers for *Linum suffruticosum* based on a genomic library developed with new generation sequencing, and tested their transferability to the sister species *L. tenuifolium*. The 12 SSR markers amplified and showed high levels of polymorphism in the seven populations tested for each evaluated species. All microsatellite regions were deposited in NCBI Genbank (Table 1).

In *L. suffruticosum*, the number of alleles per locus per population (*A*) ranged from 2 to 16, with a mean of 6.7; the observed heterozygosity (*H*_O_) ranged from 0.09 to 1, with a mean of 0.83; and the expected heterozygosity (*H*_E_) ranged from 0.45 to 0.9, with a mean of 0.69 (Table 2). In each population, four to nine loci deviated significantly from Hardy–Weinberg equilibrium after Bonferroni correction, and two to four loci showed presence of null alleles (Table 2). In *L. tenuifolium*, the number of alleles per locus per population (*A*) ranged from 2 to 10, with a mean of 5.1; the observed heterozygosity (*H*_O_) ranged from 0 to 1, with a mean of 0.77; and the expected heterozygosity (*H*_E_) ranged from 0.05 to 0.88, with a mean of 0.62 (Table 3). In each population, five to twelve loci deviated significantly from Hardy–Weinberg equilibrium after Bonferroni correction, and two to four loci showed presence of null alleles (Table 3). We found high levels of genetic diversity and significant deviations from Hardy–Weinberg equilibrium. These are congruent with the inherent outcrossing of the three-dimensional heterostylous *L. suffruticosum*, as well as with the potential hybridization between the two taxa in the analysed populations.

**Table 2:** Results of genotyping in populations of *Linum suffruticosum*. Localities EO35, EO36 and G8 were mixed with *L. tenuifolium*

Locality	EO35				EO36				EO5				107JAM				G12				G19				G8			
Locus	N	A	He	Ho	N	A	He	Ho	N	A	He	Ho	N	A	He	Ho	N	A	He	Ho	N	A	He	Ho	N	A	He	Ho
Ls_1145191	22	8	0.775	1.0	22	7	0.788	1.0	20	6	0.686	1.0*	19	8	0.765	1.0	24	8	0.774	1.0*	22	7	0.714	1.0*	23	9	0.824	0.909
Ls_1169143	22	6	0.8	1.0*	22	7	0.839	0.952*	20	3	0.711	1.0*	19	4	0.82	1.0	24	6	0.806	1.0	22	7	0.773	1.0	23	9	0.814	0.957*
Ls_1178187	22	6	0.539	0.909*	22	5	0.5	1.0*	20	2	0.668	0.85*	19	3	0.589	0.895	24	6	0.518	0.917*	22	5	0.619	0.909	23	5	0.725	1.0
Ls_144692	22	5	0.788	1.0*	22	4	0.737	0.909*	20	5	0.547	1.0*	19	2	0.488	0.842	24	7	0.688	0.917*	22	7	0.56	0.545	23	7	0.723	1.0*
Ls_246481	22	11	0.848	0.909	22	13	0.673	0.7	20	6	0.656	0.8	19	6	0.687	0.579§	24	11	0.745	0.792	22	11	0.738	0.81	23	12	0.729	0.818
Ls_337128	22	4	0.84	0.682§	22	2	0.902	0.429*§	20	4	0.759	0.632§	19	3	0.744	0.632§	24	3	0.903	0.826§	22	3	0.857	0.591* §	23	6	0.88	0.636§
Ls_37372	22	6	0.567	0.227	22	6	0.62	0.286*§	20	3	0.461	0.3§	19	2	0.69	0.263*§	24	5	0.448	0.273§	22	7	0.598	0.318* §	23	6	0.608	0.091*§
Ls_395648	22	9	0.752	0.955	22	7	0.844	0.773	20	5	0.515	0.8	19	6	0.56	0.421§	24	9	0.742	0.917*	22	8	0.652	0.955*	23	5	0.831	0.955
Ls_421659	22	10	0.714	1.0	22	16	0.71	1.0*§	20	7	0.635	1.0*	19	8	0.499	0.947	24	16	0.674	0.958*	22	13	0.739	1.0*	23	12	0.732	0.909*
Ls_807222	22	8	0.725	0.682*§	22	9	0.735	1.0*	20	8	0.58	1.0*	19	8	0.644	1.0*	24	7	0.741	0.87*	22	9	0.654	0.636	23	5	0.777	0.857*
Ls_889692	22	7	0.605	0.545*§	22	11	0.704	1.0*	20	3	0.5	1.0*	19	5	0.597	1.0*	24	7	0.66	0.75*	22	6	0.667	1.0*	23	8	0.708	1.0*
Ls_9438	22	6	0.707	1.0*	22	5	0.698	1.0*	20	5	0.635	1.0*	19	2	0.499	0.947*	24	6	0.674	1.0*	22	9	0.735	1.0*	23	6	0.741	0.955*

N = successfully amplified individuals; A = number of alleles; He = expected heterozygosity; Ho = observed heterozygosity

*Significant deviation from Hardy-Weinberg equilibrium after Bonferrroni correction (P < 0.005)

§Significant possibility of the presence of null alleles

**Table 3:** Results of genotyping in populations of *Linum tenuifolium*. Localities EO35, EO36 and G8 were mixed with *L. suffruticosum*

Locality	EO35				EO36				G16				G26				li-17-02				Spot					G8			
Locus	N	A	He	Ho	N	A	He	Ho	N	A	He	Ho	N	A	He	Ho	N	A	He	Ho	N	A	He	Ho		N	A	He	Ho
Ls_1145191	22	9	0.811	1.0*	22	8	0.773	0.682*§	22	8	0.783	1.0*	18	6	0.637	0.944*§	20	4	0.76	0.9*	22	7	0.692	0.955	*	24	9	0.76	1.0*
Ls_1169143	22	2	0.397	0*§	22	4	0.666	0.571*§	22	3	0.447	0.136*§	18	2	0.313	0.278	20	2	0.643	0.105	22	4	0.462	0.409	*§	24	2	0.375	0*§
Ls_1178187	22	4	0.698	1.0*	22	5	0.739	1.0*	22	4	0.676	1.0*	18	2	0.5	1.0	20	2	0.584	1.0*	22	4	0.583	1.0	*	24	5	0.702	1.0*
Ls_144692	22	6	0.746	0.955*	22	6	0.765	1.0*	22	6	0.711	1.0*	18	3	0.526	1.0*	20	5	0.57	0.7	22	6	0.621	1.0	*	24	5	0.765	0.833*
Ls_246481	22	8	0.749	0.909*	22	8	0.825	1.0	22	8	0.838	1.0*	18	8	0.748	1.0*	20	7	0.564	1.0	22	4	0.583	1.0	*	24	9	0.816	1.0
Ls_337128	22	3	0.522	0.955*	22	4	0.561	0.955*	22	6	0.679	1.0*	18	3	0.593	0.889	20	3	0.5	0.3*§	22	3	0.598	0.857	*	24	3	0.284	0.333
Ls_37372	22	5	0.714	1.0*	22	6	0.803	0.955*	22	5	0.729	1.0*	18	4	0.576	1.0	20	5	0.371	0.95*	22	5	0.635	1.0	*	24	5	0.727	1.0*
Ls_395648	22	4	0.697	0.955*	22	8	0.789	0.909	22	4	0.712	1.0*	18	6	0.73	1.0	20	6	0.1	0.85	22	5	0.674	0.955	*	24	6	0.672	0.917*
Ls_421659	22	2	0.127	0.136	22	5	0.249	0.091*	22	4	0.551	0.273*§	18	3	0.285	0.333	20	2	0.049	0.05	22	3	0.129	0.136		24	3	0.484	0.333§
Ls_807222	22	6	0.758	0.955*	22	7	0.765	0.75*§	22	7	0.768	0.955*	18	5	0.636	0.944§	20	4	0.686	0.947*	22	6	0.688	0.864	*	24	6	0.644	0.667*
Ls_889692	22	5	0.683	0.455§	22	10	0.879	0.714*§	22	5	0.747	0.409*§	18	5	0.622	0.389*	20	5	0.686	0.55*§	22	5	0.661	0.818	*	24	6	0.682	0.75
Ls_9438	22	8	0.754	0.955*	22	6	0.765	1.0*	22	6	0.709	0.955*	18	3	0.526	1.0*	20	5	0.678	0.7	22	5	0.62	1.0	*	24	6	0.769	0.833*

N = successfully amplified individuals; A = number of alleles; He = expected heterozygosity; Ho = observed heterozygosity

*Significant deviation from Hardy-Weinberg equilibrium after Bonferrroni correction (P < 0.005)

**§**Significant possibility of the presence of null alleles

These SSR markers will be a useful tool to investigate the mating patterns within and between *L. suffruticosum* and *L. tenuifolium* in their contact zone, and patterns of gene flow and spatial genetic structuring among and within pure and mixed populations. The genus *Linum* has been the object of renewed attention for the study of heterostyly, from macroevolutionary patterns [5, 6] to finer scale processes within or across *Linum* species and populations [2, 29], and polyploidy [25, Valdés et al., under review]. Given the full transferability success shown, these SSR markers could potentially be applied to other *Linum* species for studies of ecological genetics.

## Supplementary Information

Below is the link to the electronic supplementary material.

Supplementary material 1 (DOCX 17.6 kb)

## Data Availability

The selected SSR sequences are publicly available on GeneBank under the corresponding accession codes in Table 1.
