# Supplementary material for: Development of microsatellite markers for sister species Linum suffruticosum and Linum tenuifolium in their overlapping ranges
Source: Mol Biol Rep. 2023 Jul 17;50(9):7927–33. doi: 10.1007/s11033-023-08471-9 (PMC10460739; doi:10.1007/s11033-023-08471-9)
Supplement: Supplementary file 1 — Supplementary material 1 (DOCX 17.6 kb) [file 11033_2023_8471_MOESM1_ESM.docx]

**Molecular biology reports**

**Supplementary Information for the article:**

**DEVELOPMENT OF MICROSATELLITE MARKERS FOR SISTER SPECIES *LINUM SUFFRUTICOSUM* AND *LINUM TENUIFOLIUM* IN THEIR OVERLAPPING RANGES**

Erika Olmedo-Vicente ^1^, Aurélie Désamoré ^2^, Violeta I. Simón-Porcar ^1*^, Tanja Slotte ^2^ and Juan Arroyo ^1^

1 Department of Plant Biology and Ecology, University of Seville, Seville, Spain

2 Department of Ecology, Environment and Plant Sciences, Science for Life Laboratory, Stockholm University, Stockholm, Sweden

*Corresponding author’s e-mail: [violetasp@us.es](mailto:violetasp@us.es)

**ORCID IDs**

Violeta I. Simón-Porcar 0000-0003-4024-2824

Tanja Slotte 0000-0001-6020-5102

Juan Arroyo 0000-0003-4749-2519

**Supplementary Information 1.** Locality information for populations of *Linum suffruticosum* (*Ls*) and *L. tenuifolium* (*Lt*) used in this study. Status refers to the co-occurrence of both species in the same site (mixed) and their occurrence alone (pure). N: number of individuals sampled.

| **Species** | **Status** | **Code** | **Locality** | **Lat** | **Long** | **m asl** | **N** |
| --- | --- | --- | --- | --- | --- | --- | --- |
| *Ls* | pure | 107JAM | Pedraforca, Spain | 42.2236 | 1.7261 | 1265 | 19 |
| *Ls* | pure | EO5 | Pardailhan, France | 43.4332 | 2.8163 | 601 | 20 |
| *Ls* | pure | G12 | Moulès-et-Baucels, France | 43.9445 | 3.7513 | 211 | 24 |
| *Ls* | pure | G19 | Pahlers, France | 44.5204 | 3.3048 | 702 | 22 |
| *Ls* | mixed | EO35 | Lanuejols, France | 44.1330 | 3.3548 | 877 | 22 |
| *Ls* | mixed | EO36 | Alp, Spain | 42.3525 | 1.8419 | 1295 | 22 |
| *Ls* | mixed | G8 | Courtougou, France | 43.7386 | 3.8683 | 81 | 23 |
| *Lt* | pure | Li_17_ 2 | Occitane, Orban, France | 43.8456 | 2.0756 | 209 | 20 |
| *Lt* | pure | Espot | Espot, Spain | 42.5702 | 1.1071 | 1171 | 22 |
| *Lt* | pure | G16 | Saint Maurice de Navacelle, France | 43.8824 | 3.4983 | 638 | 22 |
| *Lt* | pure | G26 | Saint Pierre des Tripiers, France | 44.2059 | 3.2901 | 509 | 18 |
| *Lt* | mixed | EO35’ | Lanuejols, France | 44.1332 | 3.3554 | 877 | 22 |
| *Lt* | mixed | EO36’ | Alp, Spain | 42.3536 | 1.8418 | 1296 | 22 |
| *Lt* | mixed | G8’ | Courtougou, France | 43.7386 | 3.8683 | 81 | 24 |
